# CO_2_ laser-assisted sclerectomy surgery for secondary open-angle glaucoma after vitrectomy

**DOI:** 10.3389/fmed.2024.1429791

**Published:** 2024-07-30

**Authors:** Zheng Li, Ao Wang, Mingqiong Zhu, Na Zhou, Li Liu, Qiaolian Li, Guoping Kuang

**Affiliations:** ^1^Department of Ophthalmology, The First People’s Hospital of Chenzhou, Chenzhou, Hunan, China; ^2^Department of Ophthalmology, The Affiliated Chenzhou Hospital, Hengyang Medical School, University of South China, Chenzhou, Hunan, China; ^3^Shenzhen Aier Eye Hospital affiliated to Jinan University, Shenzhen, Guangdong, China

**Keywords:** secondary glaucoma, post-vitrectomy, CO2 laser, deep sclerectomy, PAS, Nd:YAG laser

## Abstract

**Purpose:**

To explore the efficiency and safety of carbon dioxide (CO_2_) laser-assisted sclerectomy surgery (CLASS) in Chinese patients with glaucoma secondary to vitrectomy.

**Methods:**

This retrospective study consisted of 16 eyes from 16 patients with glaucoma secondary to vitrectomy who underwent CLASS and were followed up for 12 months. Main outcome measures included preoperative and postoperative intraocular pressure, best corrected visual acuity (BCVA), number of anti-glaucoma medications, and postoperative surgical success rate and complications.

**Results:**

The postoperative IOP and number of anti-glaucoma medications used at all follow-up time point were significantly lower than those preoperatively. The difference in BCVA was not significant before and after surgery. The main complicatios were peripheral anterior synechiae (PAS) and scleral reservoir reduction, which were controlled after neodymium-doped yttrium aluminum garnet (Nd:YAG) laser, 2 (12.50%) patients underwent re-operation. The complete and total success rates at 12 months were 68.75% and 87.50%, respectively.

**Conclusion:**

CLASS is a safe and effective procedure for Chinese patients with glaucoma secondary to vitrectomy. PAS and scleral reservoir reduction is a major contributor to postoperative IOP elevation, and trabecular minimally invasive perforation with the Nd:YAG laser is effective in lowering IOP and increasing scleral cistern volume.

## 1 Introduction

High intraocular pressure (IOP) following vitrectomy is a frequent occurrence in clinical settings, with reported incidence rates in the literature ranging from 25.7 to 56.0% (1, 2). The etiology and pathogenesis of high IOP exhibit complexity and diversity. The majority of high IOP cases occur in the early postoperative stage, primarily due to factors such as the injection of excessive or high-concentration expansive gas, over-injection of silicone oil, inflammation, hemorrhage, and glucocorticoid sensitivity (1). The incidence of late ocular hypertension following surgery is relatively lower compared to the early stage, yet it remains occult and can have a significant impact on visual function. The etiologies of middle and late stage open-angle glaucoma primarily comprise silicone oil emulsification, elevated resistance of aqueous humor outflow resulting from glucocorticoids in the trabecular meshwork, and neovascular glaucoma during the open-angle phase. On the other hand, the etiologies of middle and late stage angle closure glaucoma encompass pupil block, peripheral anterior synechiae induced by chronic inflammation, and neovascular glaucoma in the angle closure phase.

The treatment of secondary glaucoma following vitrectomy is typically challenging (3). When medications prove ineffective in controlling IOP or are not well-tolerated due to long-term side effects, surgical intervention frequently becomes necessary. Nevertheless, traditional surgical approaches are plagued by a low success rate, numerous complications, and a poor prognosis, categorizing them as refractory glaucoma cases. During routine filtration surgery, the vitreous cavity following vitrectomy is filled with liquid, Penetrating the ocular wall may result in a sudden drop in IOP, collapse and deformation of the eyeball, and even pose risks such as explosive suprachoroidal hemorrhage and choroidal detachment. Furthermore, vitreoretinal surgery can disturb the conjunctiva and trigger a postoperative inflammatory reaction, thereby increasing the risk of long-term filtration bleb scarring, which may ultimately lead to the failure of the filtration surgery. Hence, non-filtering bleb-dependent and non-penetrating glaucoma surgery could be a viable approach for the management of refractory glaucoma post vitrectomy (4, 5). Hence, non-penetrating glaucoma surgery could be a viable approach for the treatment of refractory glaucoma post vitrectomy.

There has recently been renewed interest in non-penetrating deep sclerectomy (NPDS) for glaucoma. NPDS reduced many potential vision-threatening complications by preserving the integrity of the trabeculo-Descemet membrane (TDM) and stabilize the anterior chamber (AC), and improved the safety of conventional filtering procedures (6, 7). However, the NPDS procedure is technically difficult to perform manually, which has limited its popularity. CLASS is an improved version of NPDS that uses a CO2 laser, which is precise and easily strips the deep sclera, unroofs the Schlemm’s canal (SC) and leaves the TDM thin enough for aqueous humor percolation. Perforations can be avoided by a CO2 laser because of its unique characteristics and effectiveness in ablating only dry tissues.

CLASS can increase drainning aqueous humor through trabecular meshwork, superior choroidal space, and other routes without bleb dependency, safely reduce IOP, and avoid the common complications associated with filtration surgery. Several studies have confirmed that CLASS is safe and effective for treating primary open angle glaucoma (POAG) with its unique advantages of CO_2_ laser (8, 9), while only a few studies have reported the application of some special types of glaucoma such as primary congenital glaucoma, uveitic glaucoma, pseudoexfoliative glaucoma and a case of refractory chidhood glaucoma (6, 10–12). In this study, patients with secondary open-angle glaucoma after vitrectomy who met the inclusion criteria (whose IOP was not better controlled with medications) were treated with the CLASS surgical approach, and the postoperative outcomes were evaluated.

## 2 Materials and methods

### 2.1 Participants

This is a retrospective study. A total of 21 cases (21 eyes) that met the inclusion and exclusion criteria were collected between January 2021 and January 2022, of which 5 cases had incomplete data, so 16 eyes were included in this study. All procedures adhered to the tenets of the Declaration of Helsinki and were approved by the hospital’s ethics committee (No. 2022024). Written informed consent was provided by all patients before joining the study.

All patients underwent vitrectomy and laser photocoagulation of the retina, and the original vitreous cavity filler was removed. Among them, the primary diseases were central retinal vein occlusion (3 eyes, 18.75%), proliferative diabetic retinopathy (10 eyes, 62.50%), and rhegmatogenous retinal detachment (3 eyes, 18.75%). Intraocular lens in 4 eyes, aphakia in 3 eyes. The vitreous cavity was filled with silicone oil in 7 eyes and with filtered air in 9 eyes. The silicone oil retrieval was performed at the longest time of 14 months and the shortest of 3 months from the time of this surgery, with an average months of 7.26 ± 1.05.

Inclusion criteria: (1) The vitreous cavity filling had been removed and the retina was flat, but the IOP was elevated, which remained at >21 mmHg with the application of maximum tolerable anti-glaucoma medication, glaucomatous optic nerve morphology, and progressive visual field (VF) loss; (2) Willing to undergo surgery and sign the informed consent. (3) Preoperative gonioscopy revealed an open angle and no obstructive lesions on the trabecular meshwork surface. (4) no active inflammation for at least 3 months before the CLASS surgery.

Exclusion criteria: (1) corneal opacity or opaque refractive media that may interfere with optic nerve evaluations. (2) A history of anti-glaucoma surgery. (3) Withdrew from the study or were lost to follow-up. (4) POAG, neovascular glaucoma, pigmentary glaucoma, exfoliation syndrome, and other types of glaucoma.(5) presence of eye trauma or inflammation. (6) severe systemic disease.

### 2.2 Methods

Superficial anesthesia and bulbar subconjunctival infiltration anesthesia were used. A conjunctival flap with the superior fornix as the base was made, and Tennon’s capsule was removed. The scleral flap with size about 5 mm × 5 mm, scleral thickness of about ½, and extending 1 mm into the clear corneal zone was isolated anteriorly to fully expose the trabecular Descemetic window (TDW). After placing a 0.2–0.4 mg/mL mitomycin C (MMC) cotton sheet under the conjunctival and scleral flaps for 2–5 min (the specific time length was determined by the operating surgeon based on the patient’s condition), they were rinsed with normal saline. The sclera is ablated by a CO2 laser to make the scleral cisterna. It is recommended that the area of the sclera pool should be at least 4.0 mm × 2.0 mm, with a supporting edge of at least 0.5 mm away from the edge of the sclera flap. The initial ablation power is recommended to be 21 W, and the rectangular laser is excited perpendicular to the sclera. Ablation to reveal the uveal pigment. MMC at a recommended concentration of 0.4 mg/mL is placed at the bottom of the deep sclera pool, and the duration of exposure depends on the patient’s condition. It is recommended to remove the cotton piece after 30 s to 2 min and rinse it thoroughly. CO_2_ laser ablation of corneoscleral rim site, TDW, arc ablation was selected with ablation size at least 4.0 mm × 1.0 mm, until the Schlemm’s canal outer wall was opened and visible aqueous humor percolated, which absorbed the CO_2_ laser, preventing deep ablation, leaving a thin corneoscleral trabeculae and uveal trabecular meshwork tissue, followed by 10–0 polypropylene suture scleral flap and closed suture conjunctival flap.

If the intraoperative IOP was still >30 mmHg, a paracorneal incision puncture was performed intraoperatively before the ablation of Schlemm’s canal, slowly lowering the IOP to <30 mmHg.

Postoperatively, prednisolone acetate eye drops were applied for 1 month, every 2 h for the first 3 days, later changed to 1 day four times, and then gradually tapered according to the resolution of the inflammatory reaction for 3 weeks, followed by 1% pilocarpine eye drops for four times 1 day for 3 months.

### 2.3 Outcome measures

Patients were followed up for 1 day, 1 week, 1 month, 3 months, 6 months, and 12 months after surgery for ocular conditions, including IOP (Goldmann applanation tonometry), fundus examination, BCVA (described in LogMAR form), inflammatory reactions in the anterior segment of the eye, the number of anti-glaucoma drugs, and complications. UBM examination was required at 1, 3, 6, and 12 months after surgery.

If the IOP was >21 mmHg postoperatively and the UBM showed significant narrowing of the scleral cistern, 0.2 mL of 5-fluorouracil (5-FU, 25 mg/mL) solution was administered subconjunctivally and episclerally. Laser goniopuncture (LGP) was performed if the effect was poor, considering insufficient filtration of the aqueous humor through the trabecular meshwork and TDW. If the IOP was still >21 mmHg and UBM demonstrated peripheral anterior synechiae (PAS) to TDM, a laser peripheral iridectomy was indicated.

### 2.4 Success criteria

Complete success: 5 mmHg < IOP < 18 mmHg, IOP drop ≥20%, and no need for IOP-lowering medication or re-operation postoperatively. Conditional success: 5 mmHg < IOP < 18 mmHg, IOP reduction ≥20%, and the need for IOP-lowering medication postoperatively.

### 2.5 Statistical analysis

Descriptive statistical results are presented as mean ± standard deviation (SD) or median and range. Gender was assessed with the chi-squared test. Data normality was tested using the Shapiro–Wilk test. Nonparametric tests were applied accordingly when the normal distribution is not satisfied. Quantitative variables were compared using the paired-samples *t*-test (if normally distributed) or the Wilcoxon signed-rank test (otherwise). A *p*-value <0.05 was considered statistically significant. Statistical analysis was performed using SPSS 21.0.

## 3 Results

### 3.1 Baseline characteristics and changes in visual acuity

A total of 16 patients (16 eyes) underwent the class procedure, including 10 males (62.50%) and 6 females (37.50%). The age of the cohort was 55. 94 ± 9.84 years (range 37–72 years); IOP ranged from 33.37 ± 4. 92 mmHg (range 26–42 mmHg) preoperatively, and 3.06 ± 0.77 number (range 2–4 number) of IOP-lowering agents were used.

The preoperative logMAR BCVA was 0.80 ± 0.52, and the 1-day, 1-week, 1-month, 3-month, 6-month, and 12-month postoperative logMAR BCVA were 0.80 ± 0.49, 0.76 ± 0.46, 0.75 ± 0.45, 0.78 ± 0.53, 0.77 ± 0.52, and 0.76 ± 0.52, respectively. No significant differences were detected in BCVA between before and after surgery (*H* = 0.156, *p* = 1.000).

### 3.2 Changes in IOP and medications

**Figure 1:**
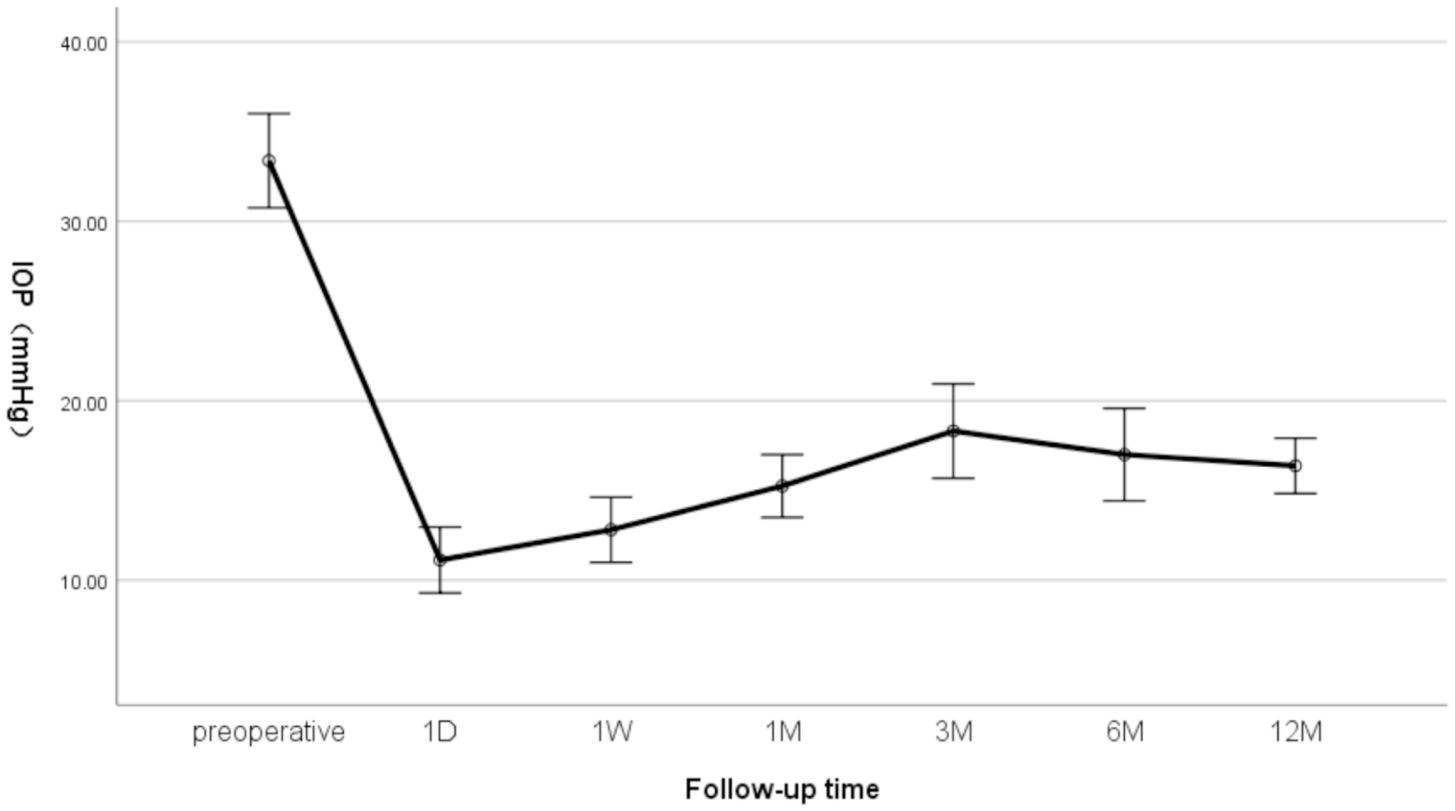
Changes in IOP at preoperative and 1 day (D), 1 week (W), and 1, 3, 6, and 12 months (M) after CLASS.

The baseline IOP was 33.37 ± 4. 92 mmHg, which decreased significantly to 15.25 ± 3.27 mmHg at 1 M after CLASS and gradually increased to 18.31 ± 4. 93 mmHg at 3 M, due to the occurrence of PAS (2 eye) and scleral cistern narrowing (2 eye). While timely Nd:YAG laser treatment, subconjunctival and scleral subvalvular injections of 5-FU solution can effectively lower and control IOP (Figure 1).

**Figure 2:**
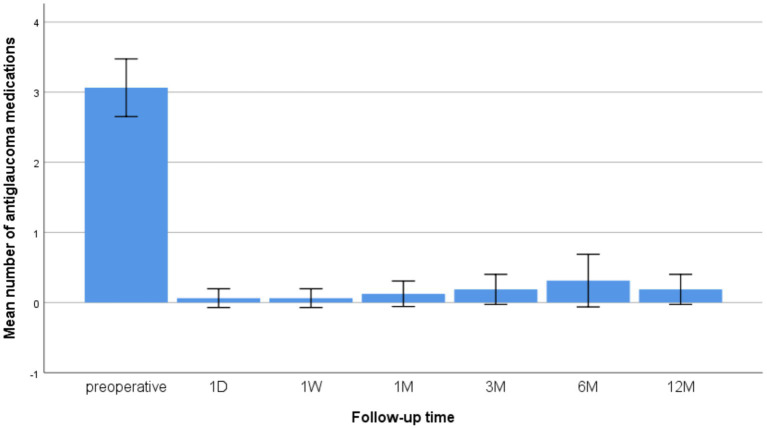
Changes in the mean number of anti-glaucoma medications at preoperative and 1 D, 1 W, and 1, 3, 6, and 12 M after CLASS.

The number of medications was 3.06 ± 0.77 before surgery. Most patients did not require anti-glaucoma medication after surgery. After 12 months of follow-up, the mean anti-glaucoma medications were only 0.19 ± 0.40 (*p* < 0.001 compared to preoperative) (Figure 2).

### 3.3 Procedural success rate

The complete success rate was 68.75%, and the conditional success rate was 87.5% at 12 months after surgery.

### 3.4 Postoperative complications

The anterior chamber hemorrhage occurred in 1 eye (6.25%), which disappeared after 1 week. The IOP was >21 mmHg in 1 eye in the first month and 2 eyes in the third month postoperatively. UBM showed PAS to trabecular meshwork Descemet’s layer (TDM), which disappeared after Nd: YAG laser treatment and IOP was lowered to the baseline value. At 3 months postoperatively, the IOP fluctuated from 20 to 22 mmHg in 2 eyes, and UBM showed scleral cistern narrowing, which improved after subconjunctival and scleral subvalvular injections of 0.2 mL 5-FU solution (25 mg/mL). During the follow-up period, the IOP was maintained 13-15 mmHg without any medication. At 6 months postoperatively, IOP was >21 mmHg in 3 eyes, UBM showed a significantly small scleral pool, the subconjunctival and scleral subvalvular injections of 5-FU were ineffective, and the IOP decreased to 16 mmHg after treatment with LGP. The scleral pool disappeared in 2 eyes at 12 months postoperatively, the IOP remained high at 35 mmHg after treatment with two types anti-glaucoma drugs, 1 eye underwent a CLASS procedure again, 1 eye underwent intraoperative perforation of the ablated area that required the conversion to trabeculectomy, and the IOP was controlled to normal levels. During the follow-up, no severe sight-threatening or irreversible complications, such as choroidal detachment, malignant glaucoma, and endophthalmitis, occurredin all patients.

By the end of follow-up, 6 individuals showed significantly smaller or disappearing scleral cisternae, including 3 eye need for 1–2 types anti-glaucoma medication, the IOP was maintained 14-16 mmHg, 2 eye underwent anti-glaucoma procedure again. The difference in age was statistically significant compared to patients without this complication (*t* = 2.466, *p* = 0.027) (Table 1).

**Table 1:** Comparison of patients with reduction of the scleral reservoir after CLASS.

	With the reduction of the scleral reservoir	Without reduction of the scleral reservoir	*p*-values
*N*	6	10	
Female/Male	1/5	3/7	0.551^*^
Age (years)	51.17 ± 10.16	62.00 ± 7.42	0.027^†^
Baseline IOP	34.00 ± 6.48	33.00 ± 4.08	0.708^†^

*Chi-squared test. ^†^Independent sample *t*-test.

## 4 Discussion

In this study, it is presumed that the increased IOP may be attributed to alterations in the trabecular meshwork and augmented resistance in aqueous humor outflow, which are induced by postoperative inflammation and glucocorticoid use. CLASS was applied to this specific type of refractory glaucoma secondary to open-angle glaucoma after vitrectomy (watery eye) and achieved good results, with the complete and success rates of 68.75 and 87.50%, respectively. Strikingly, no serious sight-threatening or irreversible complications, such as choroidal detachment, malignant glaucoma, and endophthalmitis, occurred throughout the procedure.

BCVA improved in 37.5% of the affected eyes at the last follow-up in our series, although the preoperative and postoperative visual acuity did not differ statistically significantly. Also, the number of anti-glaucoma drugs were significantly lower than before surgery.

CLASS is an improved version of the difficult manual procedure of NPDS that uses the unique characteristic of CO2 laser to ablate only the dry scleral tissue in a precise and efficient manner. Once the outer wall of Schlemm’s canal (SC) is opened, the CO2 laser becomes ineffective due to aqueous percolation; consequently, excessive tissue ablation is prevented and the inner wall of the SC is kept intact, avoiding penetration into the anterior chamber. This is due to the wavelength of the laser that interacts specifically with the molecules of water. CLASS with its unique aqueous humor drainage pathway, avoid the sudden drop of IOP after trabeculectomy surgery. Also, it is minimally invasive, easy to operate, has fewer complications, and has been gradually applied to some refractory glaucomas (13, 14).

The results of this study confirmed that the CLASS procedure is effective in favorably lowering surgical complications, reducing anti-glaucoma medications use and accelerating visual recovery, which is consistent with previous studies (4, 15, 16). However, details of the CLASS procedure and postoperative follow-up are essential. First, the Tennon’s capsule needs to be removed to reduce the scarring possibility in the scleral cistern. Second, the extent, depth, and energy magnitude of laser ablation are major issues in addition to wide range and high energy, which causes secondary damage and coagulation of surrounding tissues. A deep ablation depth has the potential to open the Schlemm canal’s inner wall, creating TDM microperforations, and requiring a trabeculectomy. None of the above complications occurred in this study, and our experience is that arcuate ablation opens the Schlemm canal’s outer wall. In the event of aqueous humor leakage, the energy of ablation needs to be reduced or the aqueous humor leakage site avoided, and the ablation of the surrounding tissue continued until the Schlemm canal outer wall is fully opened. Despite such improvement, postoperative complications, such as PAS seemed to be inevitable after CLASS. Zhang et al. observed a 30.0% PAS incidence in the CLASS group (15). Compared to Caucasian patients (16, 17), relatively higher rates of PAS were detected in Chinese patients. In this study, the incidence of PAS was 18.75%, significantly lower than the above-mentioned studies. A total of 3 patients experienced different degrees of PAS at 1 and 3 M postoperatively, which was the main cause of IOP elevation at the early-and middle-stage after CLASS. We also found that the variation of PAS incidence was consistent with the fluctuation of success rate, indicating that follow-up after CLASS was critical work for postoperative management.

Studies have shown that CLASS is prone to PAS after surgery, with iris incarceration, scleral cistern narrowing, and increased aqueous humor resistance via trabecular meshwork or TDW (11, 18, 19). Therefore, some studies proposed to modify the CLASS procedure, and hence laser peripheral iridectomy (LPI) and argon laser peripheral iridoplasty (ALPI) were performed 24–72 h before the operation (10, 11). None were preoperatively pretreated in this study, considering the following: first, not all patients who were not laser prophylactic preoperatively developed a PAS or iris incarceration postoperatively. This was also confirmed in the present study, wherein the incidence of PAS was only 18.75%. Second, guaranteeing the consistency of the location of preoperative laser therapy with the area of intraoperative laser ablation is difficult. The pervious and present studies confirmed that PAS could occur at any time after CLASS (11, 20); hence, regular follow-up and personalized postoperative management are more crucial for these patients than prevention.

No iris incarceration occurred postoperatively in our cases. Analyzing the reasons for the occurrence of iris incarceration, we speculated that this patient developed microperforation during the surgery, and previous laser intervention might cause small tears in the TDW, which aggravated iris incarceration. Therefore, meticulous ablation and energy adjustment were critical to avoid perforation during the surgery. Moreover, IOP was maintained at <30 mmHg preoperatively, and intraoperative anterior chamber puncture was performed to slowly reduce IOP, and the scleral flap was sutured tightly to avoid early postoperative hyperfiltration. Miotics were used routinely for ≥3 months postoperatively, and the massage of the eyeballs was avoided. Second, the local inflammatory response may accelerate iris incarceration. This group of cases consisted of patients with glaucoma secondary to vitrectomy, pre-existing fundus disease, and putative inflammatory factors in the eye from the trauma of vitrectomy surgery (21). The thermal damage to the surrounding tissues by laser ablation in CLASS intraoperatively can aggravate the inflammatory response. Therefore, postoperative anti-inflammation is critical.

Three cases in our series developed PAS in patients <45 years, consistent with previous studies that concluded that younger age is more likely to have postoperative complications in CLASS (20). The current study also showed that younger patients had a higher chance of developing significant scleral pool narrowing or even disappearance after CLASS, and the mean age of the 6 patients was (51.17 ± 10.16) years, which was lower than the mean age of our cases. Asian glaucoma patients, including Chinese, are characterized by crowded anterior chamber structures, intractable ocular hypertension, and easy scarring after surgery, especially young patients in whom postoperative wound fibrous proliferation is active. Therefore, prophylactic LPI and argon ALPI may be considered before CLASS for patients with high-risk interatrial angle and young glaucoma (10), and postoperative enhanced anti-inflammatory and close follow-up will improve the success rate of the procedure.

Nevertheless, the present study had several limitations. First, the sample size was small and the follow-up period was limited. Therefore, a prospective randomized controlled study with longer follow-up is necessary. Second, the morphological classification of the filtration bleb and the quantitative analysis of the scleral pool remain unclear in their correlation with IOP after CLASS. Despite these limitations, we distinguished PAS from iris incarceration by UBM examination and provided personalized timely interventions that were found to be effective in controlling IOP clinically.

To conclude, CLASS is a safe and effective approach for Chinese patients with secondary glaucoma after vitrectomy. It not only controls the IOP and reduces the dependence on drugs, but also has obvious advantages in the control of surgical complications and postoperative visual recovery. PAS and reduction of the scleral reservoir is a common cause of postoperative IOP elevation. A UBM-guided individualized Nd:YAG laser intervention resolves PAS at the early-middle stage after CLASS and achieves further IOP reduction as required, which helps in improving the long-term outcomes after CLASS.

## Data availability statement

The original contributions presented in the study are included in the article/supplementary material, further inquiries can be directed to the corresponding author/s.

## Ethics statement

The studies involving humans were approved by the Institutional Review Board/Ethics Committee of the First People’s Hospital of Chenzhou. The studies were conducted in accordance with the local legislation and institutional requirements. The participants provided their written informed consent to participate in this study. Written informed consent was obtained from the individual(s) for the publication of any potentially identifiable images or data included in this article.

## Author contributions

ZL: Data curation, Formal analysis, Funding acquisition, Methodology, Resources, Software, Validation, Writing – original draft. AW: Methodology, Writing – original draft, Supervision. MZ: Data curation, Formal analysis, Writing – original draft. NZ: Formal analysis, Writing – original draft. LL: Supervision, Writing – review & editing. QL: Writing – review & editing, Data curation, Formal analysis. GK: Conceptualization, Funding acquisition, Investigation, Project administration, Supervision, Writing – review & editing.
